# Screening for frailty and its association with activities of daily living, cognitive impairment, and falls among community-dwelling older adults in China

**DOI:** 10.1186/s12877-024-05173-0

**Published:** 2024-07-03

**Authors:** Dakang Ji, Haijian Guo, Shanhu Qiu, Lirong Dong, Ya Shen, Zhengkai Shen, Jinshui Xu

**Affiliations:** 1 Department of Integrated Services, Jiangsu Provincial Center for Disease Control and Prevention, Nanjing, 210009 China; 2 Department of Endocrinology, Zhongda Hospital, Institute of Diabetes, School of Medicine, Southeast University, Nanjing, 210009 China

**Keywords:** Frailty, Aging, FRAIL scale, ADL, Falls

## Abstract

**Objectives:**

Frailty is a prevalent geriatric condition that significantly impacts the health of older adults. This study aimed to examine the prevalence of frailty among older Chinese adults aged ≥ 65 years and to assess its association with adverse geriatric outcomes.

**Method:**

This study included 20,724 older adults aged ≥ 65 years in Jiangsu Province, China, utilizing a random, stratified, multistage cluster sampling approach. Frailty was assessed using the 5-item FRAIL scale. Geriatric outcomes, such as independence in activities of daily living (ADL), cognitive impairment, and frequent fall events (occurring four or more times in the preceding year), were evaluated. Logistic regression models were employed to evaluate the association between frailty and geriatric outcomes, with results presented as odds ratios (ORs) and 95% confidence intervals (CIs).

**Results:**

The mean age of the participants was 73.4 ± 6.4 years. The standardized prevalence of prefrailty and frailty was 35.2% and 10.3%, respectively. Individuals identified as prefrail or frail tended to live in rural areas, have lower educational levels, be widowed, have lower incomes, and engage in less physical activity. Prefrailty and frailty were associated with an increased risk of limitations in BADL (OR: 9.62, 95% CI: 7.43–12.46; and OR: 29.25, 95% CI: 22.42–38.17, respectively) and IADL (OR: 2.54, 95% CI 2.35–2.74; and OR: 5.19, 95% CI 4.66–5.78, respectively), positive cognitive impairment screening (OR: 1.23, 95% CI: 1.16–1.31; and OR: 1.72, 95% CI: 1.56–1.91, respectively), and frequent falls (occurring four or more times in the preceding year) (OR: 3.38, 95% CI: 2.50–4.56; and OR: 8.37, 95% CI: 6.01–11.65). The association between frailty and both limitations in BADL and falls was notably more pronounced among the younger age groups (p for interaction < 0.001).

**Conclusions:**

According to the 5-item FRAIL scale, frailty was associated with limitations in BADLs and IADLs, positive cognitive impairment screening, and recent falls among older adults living in the community. Screening for frailty in younger age groups has the potential to prevent declines in physical function and falls.

## Introduction

China has undergone rapid urbanization and is confronted with the world’s largest and most rapidly expanding elderly population. Jiangsu Province is one of China’s foremost coastal provinces, with 16.20% of the population aged ≥ 65 years according to China’s seventh census bureau as of November 2020 [1].

The concept of ‘healthy aging’ encompasses the absence of disease and the preservation of functional ability [2]. Frailty, described by Professor Fired in 2001 [3], is one of the most prevalent geriatric disorders impacting the health status of older adults. Frailty is a complex, age-related clinical syndrome characterized by a decline in physiological capability across multiple organ systems and an increased susceptibility to stress [4]. Researchers recommend assessing frailty using a variety of epidemiologically validated tools [5–7]. Substantial evidence has indicated an association between frailty and adverse outcomes, including hospitalization, falls, depression, and mortality [8–12]. The Fried Frailty Phenotype [3], the Frailty Index [5], and the Clinical Frailty Scale [13] are widely employed frailty assessment instruments. The Fried frailty phenotype assesses intrinsic abilities, including grip strength and gait speed, in older adults. The frailty index employs the cumulative deficit model to quantify the extent of frailty in older individuals. Nevertheless, the implementation of these two assessment tools in community settings poses challenges due to their time-consuming and location-specific nature. Similar to the frailty phenotype and the multiple deficit model, previous research has demonstrated the high reliability of the FRAIL scale [14] in predicting physical limitations and mortality. All three measures have clinical utility, with the FRAIL scale being the most straightforward instrument used [11, 15, 16]. The Asia-Pacific Clinical Practice Guidelines for the Management of Frailty recommend the FRAIL scale as a rapid screening tool for primary screening [17]. Frailty, as assessed by the FRAIL scale, has been associated with falls and hospitalizations in older Chinese adults [18]. Furthermore, frailty was assessed using the FRAIL scale as a reliable predictor of activities of daily living (ADL) impairment [19]. Numerous studies have demonstrated that frailty is not an irreversible condition [20–22]. Therefore, screening and addressing frailty may help mitigate adverse health outcomes among older adults [4].

The relationships between frailty, as assessed by the FRAIL scale, and health outcomes, such as declines in ADLs and incidents of falls, needs further evaluation due to the absence of a sizable sample for analysis in China [23]. Our study aimed to determine the prevalence of frailty among older adults aged ≥ 65 years in Jiangsu Province, one of the most significantly aging regions in China. Additionally, we aimed to investigate the correlation between frailty and adverse health outcomes according to demographic data to provide compelling evidence to support community-based frailty screening and interventions.

## Subjects and methods

### Design and participants

A total of 21,344 people were surveyed, of whom 620 refused the survey or provided incomplete information, resulting in a questionnaire validity rate of 97.10%. 20,724 residents aged ≥ 65 years completed the investigations based on provincial health status surveillance in Jiangsu Province, China. A multistage, random, stratified cluster sampling design was used to recruit participants, entailing the following steps: (1) Six prefecture-level cities were selected randomly: Wuxi, Changzhou, Taizhou, Lianyungang, Yancheng, and Suqian. (2) Two districts/counties were chosen randomly from each city. (3) Three townships/streets were selected randomly from the sampled districts or counties. (4) At each monitoring point, three villages or resident committees were chosen randomly. The inclusion criteria were as follows: 1) ≥ 65 years of age. 2) Participants voluntarily completed the survey after providing informed consent. 3) The older adult or their family could communicate with the investigator. The exclusion criteria were as follows: (1) Participants who were not present at their residences during the survey. (2) Participants who refused to engage or failed to complete the questionnaire. This study was approved by the Ethical Review Committee of Jiangsu Provincial Center for Disease Control and Prevention (JSJK2023–B012–02).

### Frailty assessment

Frailty was assessed using the FRAIL scale, which consists of 5 items: fatigue, resistance, ambulation, illness, and loss of weight. This instrument was developed based on the consensus of a European, Canadian, and American geriatric advisory panel. The FRAIL scale consists of five simple yes-or-no questions, with each component scoring one point. The total score ranges from 0 to 5, with higher scores indicating a greater degree of frailty. In the original version, a score of 0 indicates robustness, 1 to 2 indicates prefrailty, and ≥ 3 indicates frailty [14]. Previous evidence suggests that the Chinese version of the FRAIL scale is reliable and valid among elderly Chinese adults [24].

### Activities of daily living (ADL)

The participants’ basic activities of daily living (BADL) were assessed using an independence of daily living assessment form. The BADLs assessed the basic daily self-care activities of the participants. It includes feeding, grooming, dressing, toileting, and transferring. Each item has a detailed description of its various levels and scores, and the total score is calculated by summing all the scores, ranging from 0 to 37. The participants were categorized as either BADL independent (BADL score < 4) or as having limitations in BADL (BADL score ≥ 4).

The instrumental activities of daily living (IADL) scale developed by Lawton and Brody was used to assess IADL performance in this survey [25]. The scale consists of eight items, each with 4 levels, and any item that cannot be completed independently is considered a limitation in IADL. The IADLs evaluated more complex daily life activities associated with interactions between individuals and the external environment.

### Cognitive impairment screening

The subjective cognitive impairment screening of the participants was assessed utilizing the Cog-12, which is a modified and translated iteration of the Ascertain Dementia 8 (AD8) questionnaire. The original version of the AD8 questionnaire included eight yes-or-no questions [26], while the cog-12 included four additional questions: “Q9. Have you been encountering emotional instability or modifications in your personality? “; “Q10. Are you exhibiting unusual behavior or experiencing changes in your habits? “; “Q11. Have you been experiencing hallucinations? “; “Q12. Are you encountering difficulties in speech or communication?” In contrast to the AD8, the Cog-12 expanded the grading of scale options, with each item utilizing a 0–4 Likert scale. The accumulation of scores from the 12 items resulted in a total score ranging from 0 to 48. According to previous clinical evidence from China, a score of 6 or higher was identified as the optimal threshold for a positive result in this study [27].

The objective cognitive impairment screening of the participants was assessed utilizing the Mini-cog, which is a recommended brief cognitive evaluation tool for primary care [28, 29]. The assessment included a three-word recall task and the clock drawing test (CDT). A positive screening for cognitive impairment was indicated by a delayed word recall score of 0 (out of 3) or if the delayed word recall score was 1 or 2, alongside an abnormal CDT result.

### Falls

Fall conditions were assessed by a self-reported question: “How many times have you fallen in the preceding year?” (No; 1 ∼ 3 times; more than 4 times).

### Covariates

The respondents’ personal information was collected by trained staff using a questionnaire based on Android tablet devices. The sociodemographic data included the respondents’ sex, age, marital status, place of residence, cohabitants, annual personal income (categorized as high income for the upper quartile, low income for the lower quartile, and middle income for the remainder), and level of education. The behavioral characteristics included tobacco use, alcohol consumption, and physical activity (frequency of exercise per week, more than 30 min per session).

### Quality control

The data were gathered using an Android tablet by primary care physicians or registered nurses who underwent prior training and testing before utilizing the device. Our electronic survey system includes pre-specified system checks and edit checks for data control. At the same time, the survey equipment is equipped with an automatic recording system that continues until the respondent completes the survey and uploads both the survey content and the recording to the data center. Additionally, a quality control team was assembled. The team’s roles and responsibilities were explicitly defined, including questionnaire verification and content revalidation.

### Statistical analysis

Descriptive analyses were performed on all baseline variables, comparing participants by frailty status. For statistical evaluations of demographic and behavioral characteristics, one-way analysis of variance was used for continuous variables, and two-sided χ^2^ was used for categorical variables. The standardization of prevalence was based on the sex and age composition of the population sample survey data in the *Jiangsu Statistical Yearbook 2021* [30]. The risk factors influencing frailty were analyzed using a multivariate logistic regression model. Logistic regression models were used to investigate the association between health outcomes and frailty. Four binary models and a multivariate model were employed to adjust for demographic and behavioral variables such as age, sex, education level, marital status, residence status, income, tobacco use, alcohol consumption, and physical activity. We use variance inflation factor (VIF) or generalized variance inflation factor (GVIF) to check for perfect multicollinearity between the independent variables in the model [31]. The strength of the relationship was assessed by the odds ratio (OR) and its 95% confidence interval (CI). We conducted stratified analyses to examine the association between frailty and adverse outcomes in different age groups and sexes, as there is no consensus on the recommended age for frailty screening and intervention. All the statistical analyses were performed using R (v4.2.1) and SPSS 24.0, and two-sided *p*-values < 0.05 were considered to indicate statistical significance.

## Results

### Prevalence of frailty by demographic status

A total of 20,724 Chinese community-dwelling seniors aged 65 years and older participated in the study; of these, the average age was 73.55 (SD = 6.43) years, 52.4% were female, and 39.05% were residing in rural areas. Using the FRAIL scale, we identified 2151 (10.38%) and 7291 (35.18%) participants as frail or prefrail, respectively, and the prevalence standardized by age and sex was 35.17% and 10.34%, respectively. A total of 1036 (5.00%) and 5498 (26.53%) patients had limitations in BADLs and IADLs. Additionally, 45.83% and 36.13% of participants had positive subjective and objective cognitive impairment respectively. Table 1 displays baseline sociodemographic parameters measured by the FRAIL scale. Individuals with prefrailty or frailty were more likely than individuals without frailty to be older, reside in rural areas, have lower educational attainment, be widowed, have a lower income, and engage in physical activity less frequently (all *P* < 0.05). Individuals with prefrailty or frailty were more likely to have limitations in BADL or IADL, positive cognitive impairment screening, or experience more fall events (all *P* < 0.05).

**Table 1:** Sociodemographic characteristics of study participants according to frailty status

Characteristics		Total (*N* = 20,724)		Non-frail (*N* = 11,282)		Pre-frail (*N* = 7291)		Frail (*N* = 2151)	*P*
		*N* (%)		*N* (%)		*N* (%)		*N* (%)	
Age	Years, (mean ± SD)	73.55 ± 6.43		72.64 ± 5.88		74.54 ± 6.86		74.98 ± 6.87	< 0.001
	65–69	6805 (32.84)		4154 (36.82)		2096 (28.75)		555 (25.80)	< 0.001
	70–74	5985 (28.88)		3446 (30.54)		1966 (26.96)		573 (26.64)	
	75–79	4100 (19.78)		2104 (18.65)		1519 (20.83)		477 (22.18)	
	≥ 80	3834 (18.50)		1578 (13.99)		1710 (23.45)		546 (25.38)	
Gender	Male	9864 (47.60)		5749 (50.96)		3225 (44.23)		890 (41.38)	< 0.001
	Female	10,860 (52.40)		5533 (49.04)		4066 (55.77)		1261 (58.62)	
Marital status	Married	16,251 (78.42)		9335 (82.74)		5385 (73.86)		1531 (71.18)	< 0.001
	Unmarried/Separation/Divorce	397 (1.92)		210 (1.86)		152 (2.08)		35 (1.63)	
	Widowed	4076 (19.67)		1737 (15.40)		1754 (24.06)		585 (27.20)	
Educational level	Primary School or below	14,641 (70.65)		7267 (64.41)		5677 (77.86)		1697 (78.89)	< 0.001
	Junior high school	4270 (20.60)		2765 (24.51)		1177 (16.14)		328 (15.25)	
	Senior high school or above	1813 (8.75)		1250 (11.08)		437 (5.99)		126 (5.86)	
Area	Urban	12,632 (60.95)		7769 (68.86)		3757 (51.53)		1106 (51.42)	< 0.001
	Rural	8092 (39.05)		3513 (31.14)		3534 (48.47)		1045 (48.58)	
Co-residence status	Living alone	2999 (14.47)		1404 (12.44)		1245 (17.08)		350 (16.27)	< 0.001
	Living with others	17,561 (84.74)		9781 (86.70)		5995 (82.22)		1785 (82.98)	
	Senior living facilities	164 (0.79)		97 (0.86)		51 (0.70)		16 (0.74)	
Income level	Low	5014 (24.19)		2015 (17.86)		2189 (30.02)		810 (37.66)	< 0.001
	Medium	10,489 (50.61)		5681 (50.35)		3766 (51.65)		1042 (48.44)	
	High	5221 (25.19)		3586 (31.79)		1336 (18.32)		299 (13.90)	
Smoking status	Ever	4261 (20.56)		2455 (21.76)		1467 (20.12)		339 (15.76)	< 0.001
	Never	16,463 (79.44)		8827 (78.24)		5824 (79.88)		1812 (84.24)	
Alcohol status	Ever	4444 (21.44)		2671 (23.67)		1444 (19.81)		329 (15.30)	< 0.001
	Never	16,280 (78.56)		8611 (76.33)		5847 (80.19)		1822 (84.70)	
Physical exercise	No	11,993 (57.87)		5508 (48.82)		4892 (67.10)		1593 (74.06)	< 0.001
	1–2 times per week	3403 (16.42)		2110 (18.70)		1017 (13.95)		276 (12.83)	
	3 or more times per week	5328 (25.71)		3664 (32.48)		1382 (18.95)		282 (13.11)	
Limitation in BADL	No	19,688 (95.00)		11,214 (99.40)		6763 (92.76)		1711 (79.54)	< 0.001
	Yes	1036 (5.00)		68 (0.60)		528 (7.24)		440 (20.46)	
Limitation in IADL	No	15,226 (73.47)		9684 (85.84)		4586 (62.90)		956 (44.44)	< 0.001
	Yes	5498 (26.53)		1598 (14.16)		2705 (37.10)		1195 (55.56)	
Cognitive impairment screening (mini-cog)	Negative	11,227 (54.17)		6888 (61.05)		3508 (48.11)		831 (38.63)	< 0.001
	Positive	9497 (45.83)		4394 (38.95)		3783 (51.89)		1320 (61.37)	
Cognitive impairment screening (cog-12)	Cog-12 scores (median, IQR)	3 (0 ∼ 8)		1 (0 ∼ 5)		5 (1 ∼ 10)		9 (3 ∼ 15)	< 0.001
	Negative	13,237 (63.87)		8539 (75.69)		3932 (53.93)		766 (35.61)	< 0.001
	Positive	7487 (36.13)		2743 (24.31)		3359 (46.07)		1385 (64.39)	
Falls (last year)	No	17,310 (83.53)		10,133 (89.82)		5728 (78.56)		1449 (67.36)	< 0.001
	1–3 times last year	3097 (14.94)		1085 (9.62)		1410 (19.34)		602 (27.99)	
	4 or more times last year	317 (1.53)		64 (0.57)		153 (2.10)		100 (4.65)	

### Factors associated with frailty

The multivariate logistic regression model revealed that age was a significant influencing factor on frailty; compared to participants aged 65–69 years, participants aged 80 years and older had ORs and 95% CIs that increased to 1.79 (1.63–1.97) for prefrailty and 1.93 (1.67–2.22) for frailty. Prefrailty, but not frailty, was significantly more prevalent in women (OR = 1.11, 95% CI = 1.03–1.20) and was significantly less prevalent among participants with higher education levels. Compared with urban participants, rural participants were more likely to be prefrail (OR = 1.74, 95% CI = 1.62–1.85) or frail (OR = 1.64, 95% CI = 1.48–1.81). Prefrailty (OR = 1.33, 95% CI: 1.20–1.46) and frailty (OR = 1.59, 95% CI: 1.38–1.83) were more common in widowed seniors. Individuals with a high income and who exercised regularly had a lower risk of prefrailty and frailty (Table 2). No perfect multicollinearity was found among the independent variables, as all variance inflation factor (VIF) values were less than 2.

**Table 2:** Factors associated with frailty from the multivariate logistic regression model

Characteristics		Pre-frail		Frail	
		OR (95% CI)	*P*	OR (95% CI)	*P*
Gender	Male	1.00		1.00	
	Female	1.11 (1.03–1.20)	0.006	1.02 (0.91–1.15)	0.752
Age groups (years)	65–69	1.00		1.00	
	70–74	1.07 (0.99–1.16)	0.076	1.15 (1.01–1.31)	0.031
	75–79	1.31 (1.20–1.43)	< 0.001	1.45 (1.26–1.66)	< 0.001
	≥ 80	1.79 (1.63–1.97)	< 0.001	1.93 (1.67–2.22)	< 0.001
Marital status	Married	1.00		1.00	
	Unmarried/Separation/Divorce	1.26 (1.00–1.59)	0.053	1.27 (0.86–1.87)	0.233
	Widowed	1.33 (1.20–1.46)	< 0.001	1.59 (1.38–1.83)	< 0.001
Educational level	Primary School or below	1.00		1.00	
	Junior high school	0.80 (0.74–0.87)	< 0.001	0.89 (0.77–1.02)	0.085
	Senior high school or above	0.78 (0.69–0.89)	< 0.001	1.00 (0.81–1.24)	0.984
Area	Urban	1.00		1.00	
	Rural	1.74 (1.62–1.85)	< 0.001	1.64 (1.48–1.81)	< 0.001
Co-residence status	Living alone	1.00		1.00	
	Living with family	1.06 (0.95–1.18)	0.318	1.27 (1.08–1.50)	0.003
	Senior living facilities	0.87 (0.60–1.24)	0.436	1.04 (0.59–1.83)	0.886
Income status	Low	1.00		1.00	
	Medium	0.67 (0.62–0.72)	< 0.001	0.51 (0.46–0.57)	< 0.001
	High	0.59 (0.53–0.65)	< 0.001	0.37 (0.31–0.43)	< 0.001
Smoking status	Never	1.00		1.00	
	Ever	0.98 (0.90–1.07)	0.700	1.25 (1.08–1.45)	0.003
Alcohol status	Never	1.00		1.00	
	Ever	1.13 (1.04–1.23)	0.004	1.44 (1.25–1.66)	< 0.001
Physical exercise	No	1.00		1.00	
	1–2 times per week	0.64 (0.58–0.69)	< 0.001	0.55 (0.48–0.63)	< 0.001
	3 or more times per week	0.60 (0.55–0.65)	< 0.001	0.39 (0.34–0.45)	< 0.001

### Associations of frailty with ADLs, cognitive function, and falls

The results indicated that prefrailty and frailty were associated with limitations in BADL (OR: 9.62, 95% CI: 7.43–12.46; and OR: 29.25, 95% CI: 22.42–38.17) and IADL (OR: 2.54, 95% CI 2.35–2.74; and OR: 5.19, 95% CI 4.66–5.78). Objective cognitive impairment screening positive was related to prefrailty (OR: 1.23, 95% CI: 1.16–1.31) and frailty (OR: 1.72, 95% CI: 1.56–1.91). Moreover, prefrailty and frailty were associated with subjective cognitive impairment screening positive (OR: 2.17, 95% CI: 2.03–2.32; and OR: 4.40, 95% CI: 3.98–4.88). Frequent falls (four or more times in the preceding year) were related to prefrailty (OR: 3.38, 95% CI: 2.50–4.56) and frailty (OR: 8.37, 95% CI: 6.01–11.65) (Table 3). No perfect multicollinearity was found among the independent variables, as all variance inflation factor (VIF) or generalized variance inflation factor (GVIF) values were less than 2.

**Table 3:** Association of frailty with BADLs, IADLs, cognitive impairment, and falls

Outcomes	FRAIL scale				
	No-frail		Pre-frail		Frail
Limitation in BADL					
Unadjusted OR (95% CI)	1.00		12.88 (9.98–16.60)		42.41 (32.67–55.02)
Base model: OR (95% CI)	1.00		11.28 (8.73–14.56)		37.23 (28.64–48.38)
Extended model: OR (95% CI)	1.00		9.62 (7.43–12.46)		29.25 (22.42–38.17)
Limitation in IADL					
Unadjusted OR (95% CI)	1.00		3.57 (3.33–3.84)		7.58 (6.85–8.37)
Base model: OR (95% CI)	1.00		3.22 (3.00–3.47)		6.92 (6.23–7.68)
Extended model: OR (95% CI)	1.00		2.54 (2.35–2.74)		5.19 (4.66–5.78)
Cognitive impairment (Mini cog)					
Unadjusted OR (95% CI)	1.00		1.69 (1.59–1.79)		2.49 (2.27–2.74)
Base model: OR (95% CI)	1.00		1.54 (1.45–1.63)		2.22 (2.02–2.45)
Extended model: OR (95% CI)	1.00		1.23 (1.16–1.31)		1.72 (1.56–1.91)
Cognitive impairment (Cog-12)					
Unadjusted OR (95% CI)	1.00		2.66 (2.50–2.83)		5.63 (5.10–6.21)
Base model: OR (95% CI)	1.00		2.42 (2.27–2.59)		5.11 (4.63–5.65)
Extended model: OR (95% CI)	1.00		2.17 (2.03–2.32)		4.40 (3.98–4.88)
Fall 1 ∼ 3 times last year					
Unadjusted OR (95% CI)	1.00		2.30 (2.11–2.50)		3.88 (3.46–4.35)
Base model: OR (95% CI)	1.00		2.21 (2.03–2.41)		3.69 (3.29–4.15)
Extended model: OR (95% CI)	1.00		1.98 (1.81–2.17)		3.26 (2.89–3.67)
Fall 4 or more times last year					
Unadjusted OR (95% CI)	1.00		4.23 (3.15–5.67)		10.93 (7.95–15.03)
Base model: OR (95% CI)	1.00		3.92 (2.91–5.26)		9.90 (7.18–13.66)
Extended model: OR (95% CI)	1.00		3.38 (2.50–4.56)		8.37 (6.01–11.65)

Notes: Base model: Adjusted for age and gender only; Extended model: Adjusted for age groups, gender, education, marital status, living status, income, lifestyle (smoking, drinking, and physical exercise)

### Stratification analyses

**Fig. 1:**
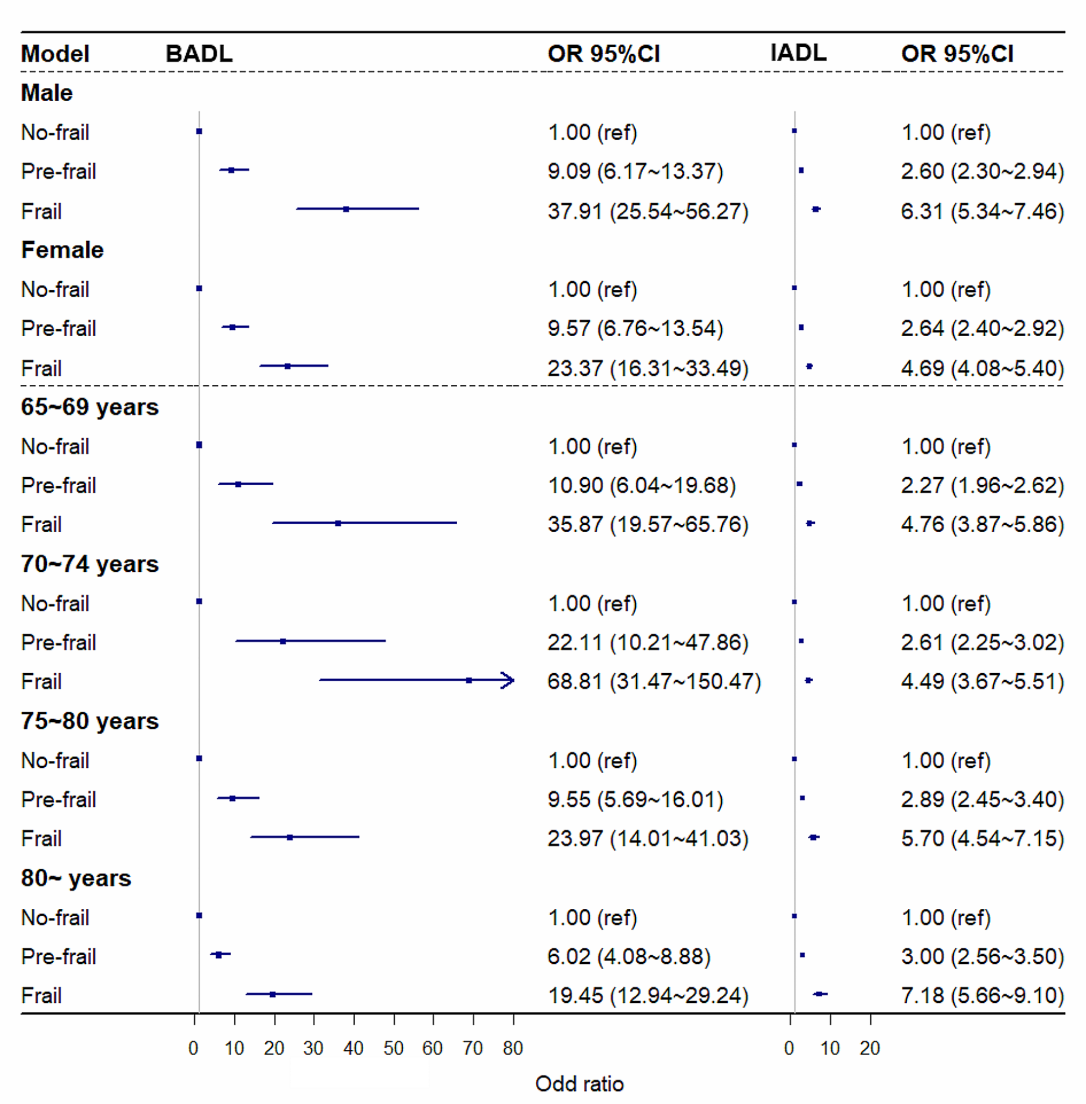
The association of frailty status with BADL and IADL among different gender and age groups Adjusted for gender, education, marital status, living status, income, and lifestyle (smoking, drinking, and physical exercise)

**Fig. 2:**
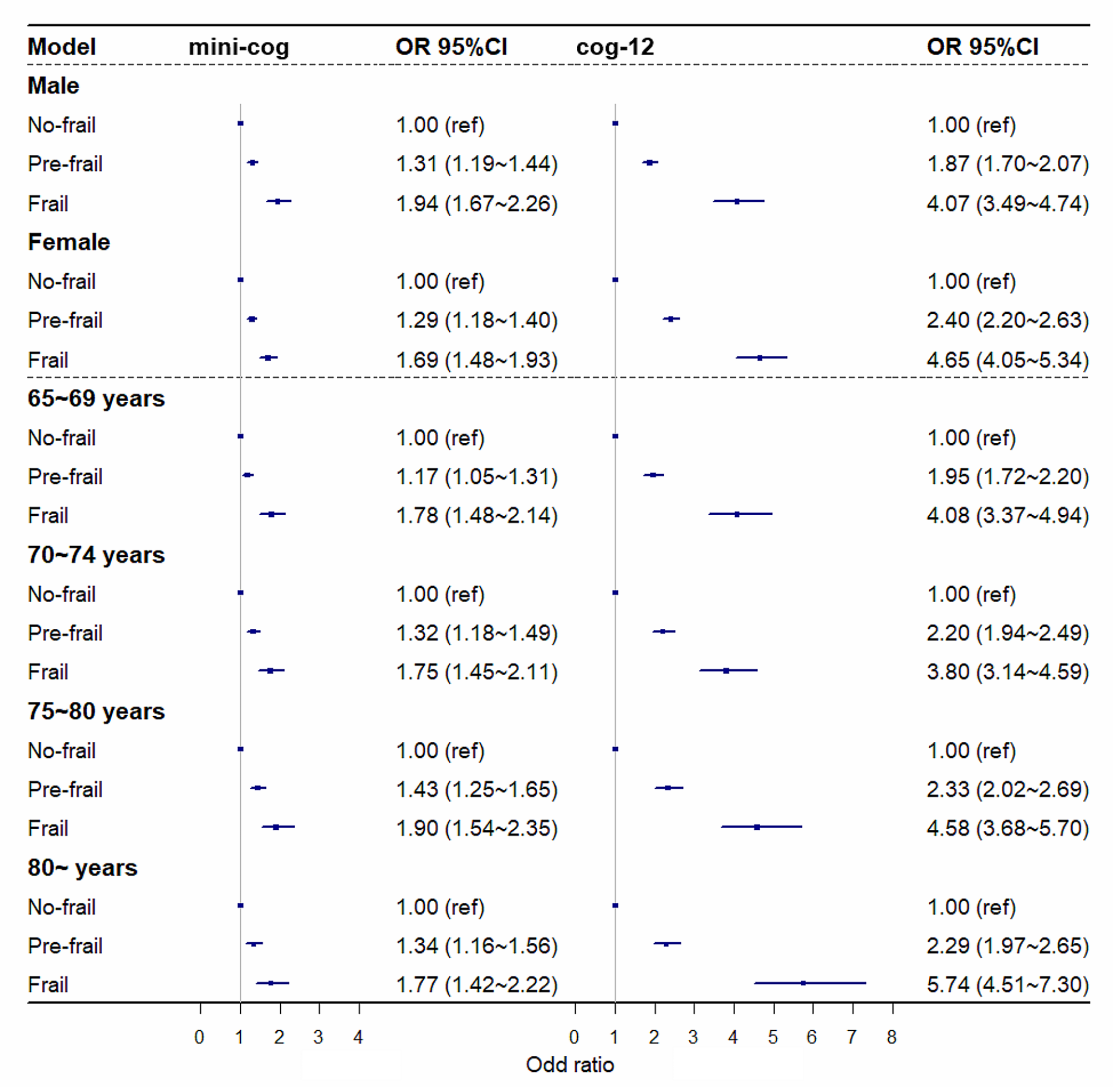
The association of frailty status with positive cognitive impairment screening among different gender and age groups Adjusted for gender, education, marital status, living status, income, lifestyle (smoking, drinking, and physical exercise)

**Fig. 3:**
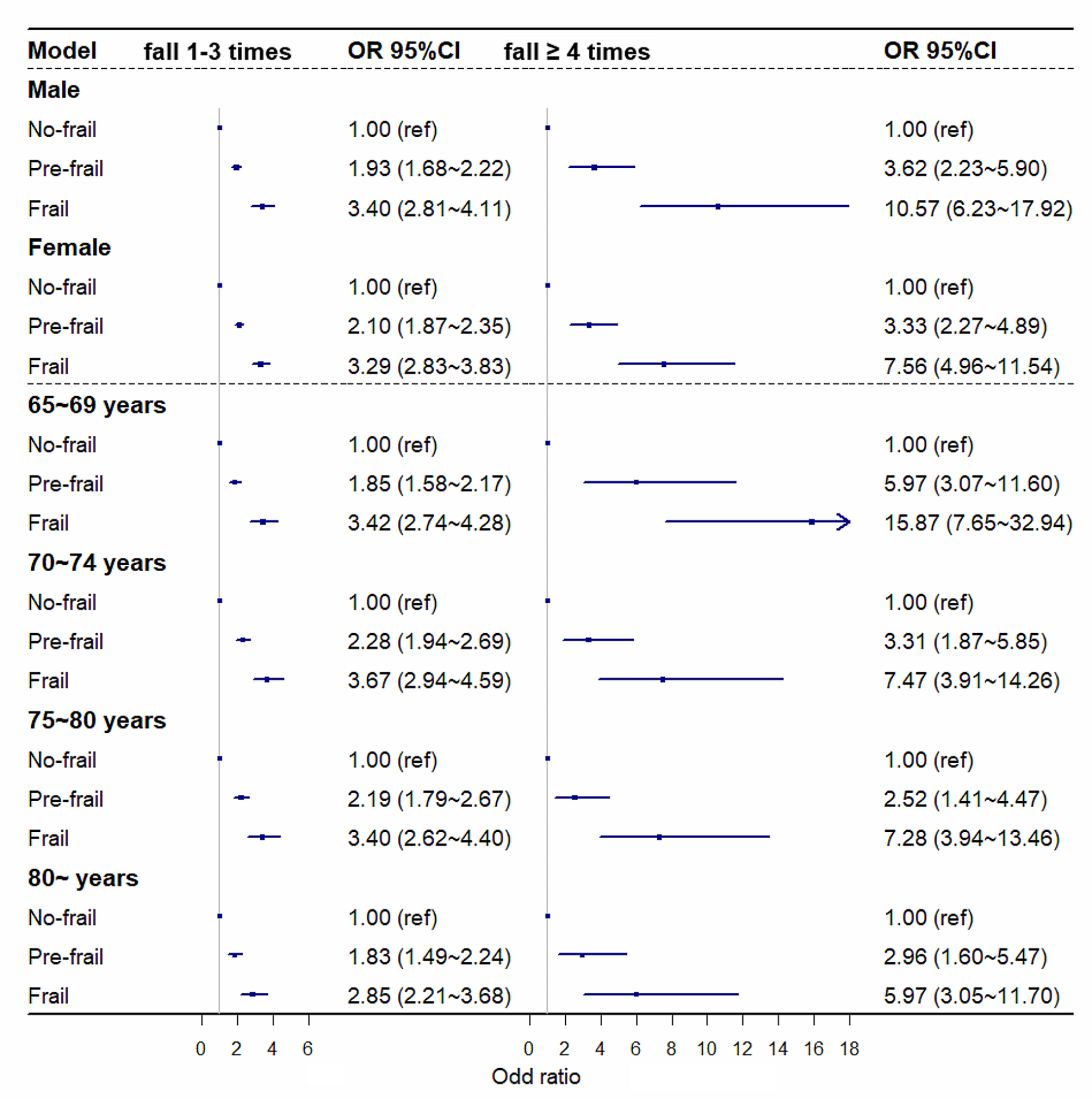
The association of frailty status with falls among different gender and age groups Adjusted for gender, education, marital status, living status, income, lifestyle (smoking, drinking, and physical exercise)

In the stratified analyses, associations between frailty and adverse outcomes were evaluated independently by sex and age. The relationships between frailty and BADL and IADL differed between sexes and age groups (all *P*_for interactions_ < 0.001). Moreover, these associations were more pronounced among males than females. Furthermore, discrepancies were observed between age groups concerning the association between frailty and BADLs, with such associations being more robust in the younger age groups than in the older age groups (Fig. 1). Additionally, the association between frailty and positive cognitive impairment screening varied across sex and age group (all *P*_for interactions_ < 0.001). The association between frailty and subjective cognitive impairment screening positive was stronger in the older age group (Fig. 2). Disparities were also detected in the association between frailty and falls across sex and age groups (all *P*_for interaction_ < 0.001), and the associations between frailty and the risk of frequent falls were more pronounced in younger age groups (Fig. 3).

## Discussion

Our study revealed that, based on the FRAIL criteria, the standardized prevalence of frailty and prefrailty was 10.34% and 35.17%, respectively, among community-dwelling adults aged 65 and older in Jiangsu Province. Despite Jiangsu Province and Shanghai being regions with significant aging populations in China, the prevalence in our research was lower than that in Shanghai, where frailty and prefrailty were reported at 16.9% and 39.5%, respectively [32]. Moreover, our results indicated a lower prevalence than that of a recent meta-analysis, which reported a frailty prevalence of 15% (95% CI: 11–21%, I^2^ = 88.4%, *P* < 0.001) among Chinese individuals aged 65 years and older based on the FRAIL scale [33].

Frailty and prefrailty were more common among women than men according to our research, which is consistent with the findings of previous studies [34]. However, in the multifactorial model, only prefrailty status was shown to be associated with sex. Our study confirmed that frailty is age-related, as has been shown in many other studies [33, 35]. In contrast to the findings of a previous multicenter study of frailty in hospitalized older adults in China [35], our research indicates that frailty is more prevalent among older adults who never drink. Using a sample of 2544 community-dwelling individuals, Gotaro Kojima et al. discovered that nondrinkers are more likely to develop frailty than people with minimal alcohol intake [36]. According to our results, regular exercise may protect against frailty, which is consistent with the findings of a recent review [37]. However, further research is needed to determine whether physical activity interventions for frailty management are feasible and cost-effective. In contrast to the findings of a cross-sectional study [32], our findings indicate that widowed older adults have a greater risk of frailty and prefrailty. Policies related to aging should prioritize frail older widowed individuals with unmet care needs.

Researchers in geriatric health have endeavored to ascertain the relationship between frailty and adverse health outcomes in older adults. Compelling evidence is crucial for assessing the advantages of screening for frailty in older adults. Our research showed that frail older people are more likely to have fallen in the past year and tend to have more falls in the past year. Reasonably, frail older adults are vulnerable to falls when they cannot rely on their physical features to compensate for their functional deficiencies. Additionally, frailty influences the prognosis for individuals who fall or who are in extended bed rest, potentially hastening the progression of frailty [38]. Our findings are consistent with a previous meta-analysis revealing a significantly elevated risk of ADL disability (frailty: OR = 9.82, 95% CI = 4.71–20.46; prefrailty: OR = 2.08, 95% CI = 1.77–2.45) and IADL (frailty: OR = 2.50, 95% CI = 1.67–3.73; prefrailty: OR = 1.74, 95% CI = 1.10–2.70) [19]. Frailty is commonly recognized as the physical condition preceding disability. Consequently, interventions aimed at preventing or slowing the progression of frailty before functional decline are essential in healthcare policy and provision. Our findings revealed a correlation between frailty and cognitive impairment in older adults. An investigation in China revealed a heightened probability of cognitive impairment among frail individuals compared to robust adults [34]. Notably, the term “cognitive frailty” has already been introduced in the last decade [39]; individuals experiencing both cognitive impairment and physical frailty exhibit a greater mortality risk than those with either condition in isolation [40]. This correlation between physical frailty and positive cognitive impairment screening provides valuable insights into the development of policies promoting healthy aging. Notably, our study revealed a more pronounced correlation between frailty and both BADLs and falls among the younger elderly age groups. This finding suggested that frailty screening in younger elderly age groups has the potential to improve the prevention of physical function decline and falls.

The FRAIL scale may serve as a viable alternative for screening for frailty in community health workers. We used a large sample size to estimate the prevalence of frailty in communities aged ≥ 65 years in a severely aging region of eastern coastal China. Our study investigated the factors influencing frailty while analyzing the association between frailty and health outcomes in community-dwelling older adults. However, due to the nature of cross-sectional studies, we were unable to determine the causal relationship between adverse outcomes and frailty or the predictive capacity of frailty. Additionally, the evaluation of frailty among participants relied on self-subject reports, potentially resulting in an underestimation of the actual prevalence of frailty.

## Conclusion

In various urban and rural areas of six cities in Jiangsu Province, the standardized prevalence of frailty and prefrailty was 10.34% and 35.17%, respectively. This study revealed significant associations between frailty and various factors, including age, widowhood, rural residence, income level, alcohol consumption, and physical activity. Furthermore, frailty was shown to be associated with limitations in BADL and IADL, positive cognitive impairment screening, and occurrence of falls in the preceding year. Frailty screening may be more effective at mitigating declines in physical function and reducing the risk of falls in younger elderly age groups. The FRAIL scale can serve as an initial screening tool for identifying frailty in older adults in the community. Effectively addressing frailty may necessitate a comprehensive geriatric assessment of the identified frail population and the implementation of specific measures aimed at mitigating the development of adverse outcomes.

## Data Availability

The datasets generated and analysed during the current study are not publicly available due to privacy of survey respondents but are available from the corresponding author on reasonable request. Data are located in controlled access data storage at Jiangsu Provincial Center for Disease Control and Prevention.
